# Stability of colon stem cell methylation after neo-adjuvant therapy in a patient with attenuated familial adenomatous polyposis

**DOI:** 10.1186/1471-230X-5-19

**Published:** 2005-06-07

**Authors:** Jung Yeon Kim, Robert W Beart, Darryl Shibata

**Affiliations:** 1 Departments of Pathology, University of Southern California Keck School of Medicine, Los Angeles, CA 90033, USA; 2 Colorectal Surgery, University of Southern California Keck School of Medicine, Los Angeles, CA 90033, USA

## Abstract

**Background:**

Methylation at certain human CpG rich sequences increases with age. The mechanisms underlying such age-related changes are unclear, but methylation may accumulate slowly in a clock-like manner from birth and record lifetime numbers of stem cell divisions. Alternatively, methylation may fluctuate in response to environmental stimuli. The relative stability of methylation patterns may be inferred through serial observations of the same colon.

**Case presentation:**

A 22 year-old male with attenuated familial adenomatous polyposis received neo-adjuvant chemotherapy and radiation prior to surgery for rectal adenocarcinoma. Colon crypt methylation patterns before and after neo-adjuvant therapy (62 days apart) were essentially identical with respect to percent methylation and diversity. Consistent with previous studies, methylation patterns recorded no evidence for enhanced colon crypt stem cell survival with a germline mutation (codon 215) proximal to the mutation cluster region of APC.

**Conclusion:**

The inability of neo-adjuvant therapy to significantly alter crypt methylation patterns suggests stem cells are relatively protected from transient environmental changes. Age-related methylation appears to primarily reflect epigenetic errors in stem cells that slowly accumulate in a clock-like manner from birth. Therefore, life-long human stem cell histories are potentially written within and may be read from somatic cell epigenomes.

## Background

Recent studies reveal methylation of certain CpG rich sequences increases with age in normal human colon [1,2]. These CpG rich sequences, like most CpG islands [3], are unmethylated at birth and appear to somatically accumulate cytosine methylation over many years. The etiology of such age-related methylation is uncertain, but one possible mechanism is random somatic errors during DNA replication [4]. By this mechanism, methylation would inherently increase with cell division because the CpG rich sequences are initially unmethylated. Consistent with random replication errors, methylation patterns (the 5' to 3' order of methylation) differ between cells within the same colon [4] and overall levels increase with age [1,2].

**Figure 1:**
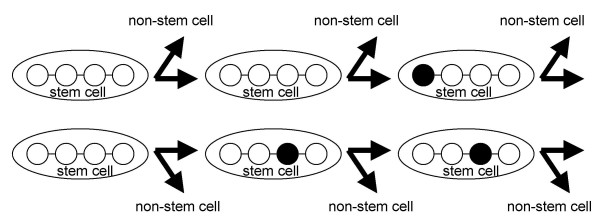
Somatic cell inheritance of methylation patterns. The tags used in this study are unmethylated at birth (unmethylated CpG sites represented by open circles, with filled circles representing methylated sites). Generally the same methylation pattern is faithfully copied and passed on to daughter cells. However, random epigenetic errors may occur, and these errors are also subsequently inherited. Methylation patterns (5' to 3' order of methylation) potentially encode numbers of divisions since birth and ancestral relationships between cells. Of note, new errors in non-stem cells will be lost during normal crypt differentiation, migration and death.

Random epigenetic errors potentially records cell histories because methylation exhibits somatic inheritance [3]. Daughter cells generally inherit the same methylation pattern as their parents, but rarely replication errors occur which are subsequently passed to progeny (Figure 1). Somatic errors can only accumulate within stem cells because non-stem cells and their errors are lost within days from normal crypt cell differentiation and migration [4,5]. Therefore, CpG rich sequences, like sequences used to reconstruct species evolution [6], potentially function as somatic epigenetic molecular clocks that can be used to reconstruct somatic cell evolution. Preliminary studies are consistent with this hypothesis because seemingly random human colon crypt methylation patterns appear to record life-long stem cell fates within crypt niches [4].

Although crypt methylation patterns from multiple different aged colons are consistent with a clock-like accumulation of epigenetic errors, environmental factors may also alter methylation. Age-related methylation levels differ between individuals of the same age [1,2,4], which may represent life-long biological differences between individuals, stochastic differences, or transient responses to environmental changes. It may be possible to distinguish between these alternatives through serial examinations of the same colon. A somatic molecular epigenetic clock predicts the amount of additional methylation is a function of the time between examinations. In contrast, if methylation is more labile, patterns may quickly change after a severe environmental insult. Here we sample methylation 62 days apart in the same colon before and after cytotoxic neo-adjuvant chemotherapy and radiation for a rectal adenocarcinoma. Methylation patterns were essentially unchanged, suggesting crypt stem cell epigenomes are relatively protected from transient environmental changes.

## Case presentation

A 22 year-old male presented with anemia. Colonoscopy revealed eight hyperplastic to adenomatous polyps ranging in size from 0.6 to 3.0 cm scattered through the colon, and a four cm, locally invasive, ulcerative rectal adenocarcinoma five cm from the anus. The clinical impression was attenuated familial adenomatous polyposis (AFAP), with a germline heterozygous truncating APC mutation at codon 215 (CAG to TAG; Gln to stop). The first sample for methylation analysis (Day 1) was a biopsy of normal appearing sigmoid colon.

The patient received neo-adjuvant chemotherapy consisting of three, two week cycles of oxaliplatin and 5-fluorouracil, with the last cycle ending two weeks before colectomy. The patient exhibited Grade 3 toxicity with mucositis, stomatitis, diarrhea, and parasthesia. He also received radiation therapy to the pelvis (2,500 centigrays over five days), ending 19 days before colectomy. The specimens obtained for analysis were outside of the primary radiation field.

Colectomy revealed approximately 50 scattered small hyperplastic to adenomatous polyps (0.1 to 0.3 cm), and a 0.7 cm ulcerative lesion at the site of the rectal adenocarcinoma containing a few residual neoplastic glands with superficial invasion of the muscularis propria. Lymph nodes were negative for metastatic carcinoma. The second specimen for analysis (Day 62) was taken from a normal appearing region of the sigmoid colon.

### Methylation analysis

Individual crypts were isolated from the first (a colonoscopic biopsy) and last (a 1–2 cm^2 ^patch of mucosa) specimens using an EDTA-containing solution as previously described [4]. DNA, extracted from individual crypts placed in microfuge tubes, was bisulphite treated (to convert C's to U's, whereas methylated-C's are unchanged), and amplified by PCR at two different CpG rich regions called CSX (nine different crypts) and BGN (eight of the same crypts). PCR products were cloned into bacterial vectors and eight clones representing individual PCR products were sequenced from each crypt [4].

**Figure 2:**
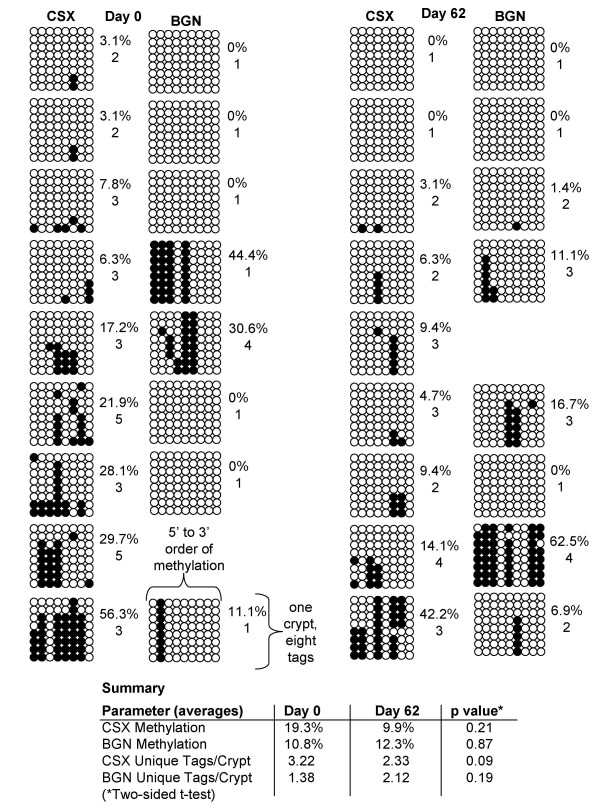
Crypt methylation before and neo-adjuvant therapy. There are eight tags per crypt with each tag arranged in a 5' to 3' horizontal order of CpG sites. CSX (eight CpG sites) and BGN (nine CpG sites) tags were sampled from eight of the same crypts. Crypt methylation patterns are summarized by percent methylation and numbers of unique tags per crypt. Methylation patterns were not significantly different before and after neo-adjuvant therapy, with averages summarize in the Table. The seemingly random methylation patterns are consistent with a model [4] in which stochastic methylation errors start to accumulate from birth.

Crypt patterns (5' to 3' order of methylation) before and after neo-adjuvant therapy were complex and seemingly random, consistent with stochastic epigenetic errors (Figure 2). The CSX (cardiac specific homeobox) sequence or "tag" is autosomal (5q34, also called Mint23 [7]) and has eight CpG sites located in the 3' untranslated region and 256 possible patterns ([2]^8 ^possible 5' to 3' orders of methylation). The BGN (biglycan) tag is on the X-chromosome (Xq28, one allele per male cell) and has nine CpG sites located just distal to the promoter and 512 ([2]^9^) possible patterns. These tags are not expressed in the colon and therefore any methylation is unlikely to confer selection [4]. Crypt methylation patterns are summarized by two statistics: percent methylation (proportion of methylated CpG sites) and diversity or numbers of unique tags per eight sampled from each crypt.

**Figure 3:**
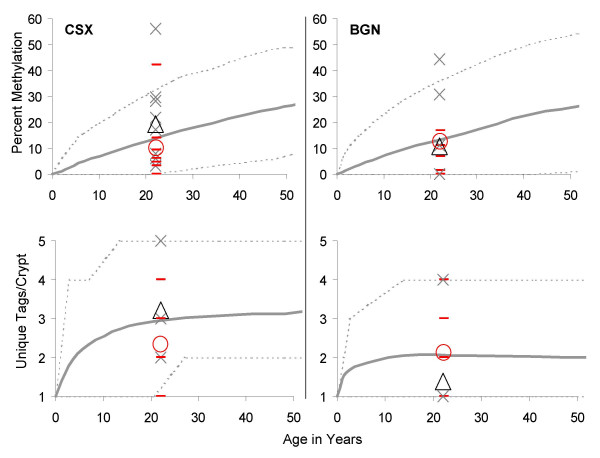
Crypt methylation tags are consistent with a clock-like stochastic accumulation of methylation errors from birth. Such a model using previously published crypt parameters [4] can be simulated on a computer to yield expected individual (dotted lines include 95% of simulated crypt outcomes) and average (solid grey line) crypt values. Little difference is expected between the initial (black X 's are individual crypt values and black triangles are average values) and final specimens (red bars are individual crypt values and red circles are average values) because by this model, methylation errors slowly accumulate from birth, and few additional changes are expected to accumulate during the 62 days between specimens.

Methylation tag values differed between crypts sampled before and after neo-adjuvant therapy, but these differences were not significant and did not follow a trend (Figure 2). Average CSX tag values were lower and BGN tags values were higher after neo-adjuvant therapy. A model [4] in which random methylation errors start to accumulate from birth is consistent with the lack of significant tag differences between serial specimens and the observed crypt variations. The model can be simulated on a computer (using previously published human colon crypt parameters [4]) to estimate average tag values and intervals that include 95% of simulated individual crypt outcomes (Figure 3). Different crypts within the same colon may have different methylation patterns because errors are stochastic and independent between cells and CpG sites. Methylation patterns before and after neo-adjuvant therapy are predicted to be similar because additional errors acquired in the 62 days between the samples are relatively insignificant compared to the 22 years since birth. Observed crypt tag values were generally consistent with the predictions of the model (Figure 3).

In addition to numbers of divisions since birth, methylation patterns (Figure 2) may also record stem cell dynamics. For example, crypt diversity or unique tags per crypt appears to reflect crypt stem cell numbers [4]. This AFAP patient appeared to have normal numbers of crypt stem cells because his crypt diversity (3.22 and 2.33 average unique CSX crypt tags) was consistent with a normal colon (Figure 3). In contrast, significantly greater crypt diversity has been observed in some familial adenomatous polyposis (FAP) patients, with an average of 3.9 unique CSX tags per FAP crypt compared to 3.0 with non-FAP crypts, which translates to about 4-fold more FAP crypt stem cells [8]. Greater numbers of crypt stem cells correlate with germline APC mutations around the mutation cluster region (MCR) that remove some but not all β-catenin binding repeats [8]. Consistent with this observation, this patient had a truncating germline mutation (Codon 215, CAG (Gln) to TAG (stop)) that removed all β-catenin binding repeats.

## Conclusion

The ability to extract historical information from normal appearing tissue is limited. However, recent advances in molecular phylogeny illustrate that genomes automatically record ancestry and time [6]. Although sequences are usually faithfully copied, errors rarely occur, which are also passed from generation to generation (Figure 1). Sequence comparisons can be used to reconstruct species evolution that occurred thousands to millions of years ago. The greater the differences between sequences, the greater the likely time since they shared a common ancestor.

Sequences comparisons depend on relatively constant error rates (a molecular clock hypothesis [6]) because time is proportional to the number of sequence differences. Although it is possible that mutation rates vary over time from environmental changes, species histories inferred from sequences are generally consistent with relatively constant mutation rates over millions of years [6]. In theory, molecular phylogeny can also be applied to somatic cell evolution because somatic cell genomes are also passed from cell to cell. Although somatic cell sequence comparisons are impractical because mutation rates are low relative to human lifetimes, epigenetic errors also potentially record ancestry because methylation exhibits somatic inheritance [3]. Epigenetic replication fidelity is less than sequence fidelity because age-related methylation is readily detected in the human colon [1,2]. Colon crypt methylation patterns have been used to infer normal human crypt niche dynamics [4], and that most directly adjacent adult crypts are not closely related because their methylation patterns differ [9]. Stem cell survival can also be inferred from methylation pattern diversity (number of unique tags). For example, increased crypt diversity and enhanced crypt stem cell survival is observed in some FAP patients with truncating germline mutations that remove some but not B-catenin binding repeats [7]. In contrast and as with this patient, germline APC mutations in the N-terminus of APC have not been associated with enhanced stem cell survival [7], and are associated with fewer polyps and AFAP [10].

A general problem with the use of sequences to reconstruct ancestry is the stochastic nature of most errors. It is difficult to prove stochastic sequence errors actually accumulate in a clock-like manner, and error rates may potentially fluctuate in response to environmental changes. Highly variable error rates would obscure ancestral information because many errors may accumulate in relatively short periods.

Although it is difficult to test a molecular clock hypothesis for species evolution, it is possible to test a somatic epigenetic molecular clock through serial examinations of the same individual. This Case Report experimentally tests the hypothesis that age-related methylation predominantly reflect stem cell errors that start to slowly accumulate from birth. Consistent with this hypothesis, methylation patterns were not significantly different after 62 days despite intervening neo-adjuvant therapy. Neo-adjuvant chemotherapy targets rapidly dividing cells and normal colon is relatively unaffected, although diarrhea (Grade 3 toxicity) may and did occur in this patient. However, the therapy did not appear to alter stem cell methylation patterns, suggesting stem cells are protected from this type of therapy. Therefore, age-related methylation appeared to be relatively stable and primarily a function of the time or number of stem cell divisions since birth. Further serial examinations with more patients and longer intervals may provide additional evidence supporting or refuting this hypothesis.

In summary, age-related methylation appears to slowly accumulate in a clock-like manner in stem cells from birth. The stochastic nature of somatic epigenetic errors requires an algorithm for interpretation, which essentially translates the sophisticated methods used to extract ancestry from species genomes to reconstruct somatic cell histories. The unseen fates of human crypt stem cells may be potentially read from anyone because methylation automatically records their autobiographies.

## Competing interests

The author(s) declare that they have no competing interests.

## Authors' contributions

Dr. Kim performed most of the experiments and helped write the manuscript. Dr. Beart provided the specimens and clinical information. Dr. Shibata analysed the data and wrote the manuscript

## Pre-publication history

The pre-publication history for this paper can be accessed here:
